# The fifth era of science: Artificial scientific intelligence

**DOI:** 10.1371/journal.pbio.3003230

**Published:** 2025-06-10

**Authors:** Nina Miolane

**Affiliations:** Department of Electrical and Computer Engineering, University of California, Santa Barbara, California, United States of America

## Abstract

Can AI become a true scientist? This Perspective explores how new technologies are reshaping scientific discovery, and why human expertise remains essential as we enter a new era of research powered by intelligent algorithms.

## Teaser

The era of artificial scientific intelligence is here. As algorithms generate discoveries at scale, what role remains for human scientists?

In 2024, artificial intelligence (AI), for the first time, helped win a Nobel Prize. DeepMind’s AlphaFold cracked one of biology’s hardest puzzles: protein folding, the challenge of predicting how a chain of amino acids twists into the intricate 3D shape that determines its function. Scientists had struggled with this problem for decades. It was crucial for medicine and drug discovery but seemed unsolvable due to the astronomical number of possible protein structures. Then AI delivered the answer.

A game-changer, no doubt. But it also raises the question: what does this mean for science and for scientists? Is traditional scientific inquiry becoming obsolete? Are we approaching a future where algorithms are the primary drivers of discovery, relegating humans to the sidelines?

**Fig 1:**
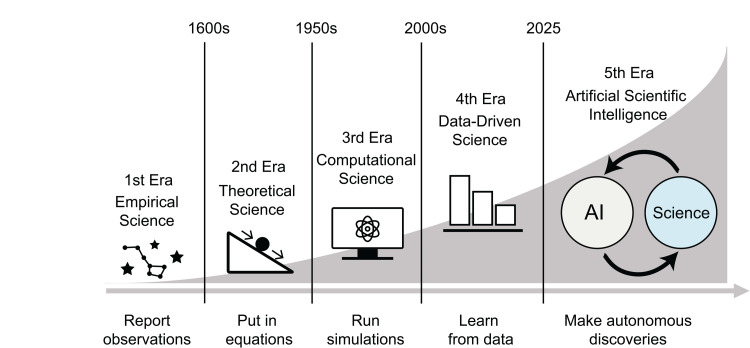
The fifth era of science: Artificial scientific intelligence. Throughout history, science has evolved through distinct eras: empirical, theoretical, computational, and data-driven. Today, it is entering the fifth era: artificial scientific intelligence. Figure credit: Nina Miolane, Haewon Jeong, and Yao Qin, University of California, Santa Barbara.

Throughout history, every breakthrough technology has redefined how discoveries were made, marking four distinct eras of science [1] (Fig 1). The first, the empirical era, relied on direct observation, as Copernicus challenged the Earth-centered view of the universe by observing the skies. The second, the theoretical era, introduced mathematics to predict nature, like Newton’s equations of motion that shaped physics for centuries. The third, the computational era, which began in the 1950s, harnessed computers to simulate complex systems, leading to Kohn and Pople’s quantum chemistry Nobel Prize. The fourth, the data-driven era of our 21st century, uses machine learning to extract patterns from vast datasets, with AlphaFold solving protein structures by learning from the protein data bank [2].

Today, we stand at the doorstep of the fifth era of science—the artificial scientific intelligence era—where companies like Google, Lila Sciences, and Sakana are unveiling AI scientists that not only assist research but drive discoveries, generate hypotheses, and test them on their own [3–5] (Fig 1). Hence, why not let AI run the show from here?

In some fields, perhaps we can. In chemistry, organic synthesis—the process of assembling complex drug-like molecules from basic building blocks—is now guided by interpretable AI models that help scientists plan each step [6]. In materials science, generative AI can design novel inorganic compounds with tailored mechanical, electronic, and magnetic properties, accelerating innovation with minimal human tuning [7]. These are domains where experimental feedback is relatively tractable, simulations are mature and the data is plentiful and structured. In short, these fields provide ideal conditions for autonomous AI exploration.

But in many other areas, letting an AI run the show today would be like sending a self-driving car down a dirt road with half a map and no GPS. AI might have the horsepower, but it still needs humans to steer it around the pitfalls of specialized scientific data. Nowhere is this clearer than in biomedical imaging, where highly curated datasets are nothing like what traditional large vision models are trained on.

First, biomedical imaging datasets are often tiny by AI standards, and for good reason: collecting them requires technical equipment and trained professionals; labeling them demands significant time and expert input; and strict privacy regulations often limit access. MedPix, a leading medical imaging database, contains just 59,000 images and the Allen Cell Feature Explorer, one of the largest publicly available collections of high-resolution 3D images of human stem cells, only around 32,000 images. That is about a thousand times fewer than what is needed for AI to perform. This is where scientists step in.

Scientists are redefining AI to do more with less, helping algorithms find meaning in images even when data are scarce. One approach involves using mathematical insights to redesign the core building blocks of neural networks. Traditional models fall apart when we strip away their layers or parameters, but these new architectures stay strong—even with just a single layer and two convolutional filters [8]—precisely because they are built to thrive on small data. And, scientists do not just bend the design of the model to fit the lack of data, they also reimagine the data ecosystem to power the model; they decide what data to collect, how to collect it, and how to weave together existing, but fragmented, specialized datasets to train AI models for a wide variety of tasks, including brain tumor classification or diabetic retinopathy grading [9].

But scientific data is not just scarce, it is often noisy. Cryo-electron microscopy (cryo-EM), a Nobel Prize-winning technology that lets us see the invisible [10]—revealing molecules at the tiniest scale—produces incredibly blurry images, where the important details are 100 times weaker than the noise. It is like trying to recognize a friend in a crowd while wearing someone else’s prescription glasses. This stands in stark contrast to the crisp, high-resolution images—like street scenes, faces, or everyday objects—that traditional AI vision models are trained on.

Yet scientists have techniques to extract meaning from even the noisiest images. In cryo-EM, they can reconstruct the 3D shapes of molecules buried in noise; for example, providing the first high-resolution images of SARS-CoV-2 during the COVID-19 pandemic [11,12]. Today, they are combining that hard-won expertise with the power of AI. One breakthrough pairs a powerful denoising module with a foundation model, enabling AI to tackle the notoriously difficult processing steps of cryo-EM images [13]. Crucially, this was only possible because scientists also applied their domain expertise to curate a high-quality dataset by cleaning, annotating, and aggregating 529 verified cryo-EM datasets into one large training set that AI could learn from.

It is clear that AI presents an enormous opportunity for science, potentially the most powerful tool we have ever had in our arsenal. But the fifth era of artificial scientific intelligence is not void of human scientists: quite the opposite. In many ways, the future of revolutionary discoveries lies in this synergy: human expertise guiding AI, and AI augmenting human expertise. It is as if we have hired the most overachieving and wildly enthusiastic intern; one who works at superhuman speed, never sleeps, and eagerly devours mountains of data. They hold exceptional potential, but without proper guidance anchored in scientific knowledge, they are more likely to set the lab on fire than to push science forward.

Instead of hoping AI will magically handle limited, noisy, specialized data, we need experts to tailor algorithms to the realities of fields like biology and medicine, and to tailor data to the new requirements of the AI technology. To enter the fifth era of science, we need to equip researchers with AI expertise, AI experts with domain knowledge, and universities with interdisciplinary programs. The labs that thrive will be those where domain experts and AI specialists work in sync or where scientists master both. The next scientific revolution will come from teams who can judiciously steer AI, knowing when to trust it, when to adjust its course, and when to drive it into uncharted territory.

## Abbreviations

AI: artificial intelligence
cryo-EM: cryo-electron microscopy
